# Books and Authors

**Published:** 1906-09

**Authors:** 


**On the Relations of Diseases of the Skin to Internal Disorders.** With observations on Diet, Hygiene and General Therapeutics. By **L. Duncan Bulkley**, A.M., M.D., Physician to the New York Skin and Cancer Hospital, Consulting Physician to the New York Hospital, Consulting Dermatologist to the Randall's Island Hospitals, to the Manhattan Eye and Ear Hospital and to the Hospital for Ruptured and Crippled, etc. Demioctavo, pp. xv-175. New York and London: Rebman Company, 1123 Broadway. (Price: $1.50).

These lectures were delivered to physicians at the New York Skin and Cancer Hospital during the month of March, 1905, and have been published in book form in compliance with numerous requests. The following synopsis indicates the scope and character of the lectures:

*Lecture I.*—1. Importance of the Skin as an Organ. 2. Predisposing and Exciting Causes of Skin Lesions. 3. Recent Recognition of Faulty Metabolism, Autointoxication and Neurotic Disturbances causing Skin Lesions. 4. Nature of Underlying Cause. 5. Broadness of True Dermatology.

*Lecture II.*—1. Digestive Disorders related to Skin Diseases. 2. Excretory Disorders related to Skin Diseases. 3. Cutaneous Disorders relating to Skin Diseases. 4. Respiratory Disturbances related to Skin Diseases.

*Lecture III.*—1. Nervous Disorders related to Skin Diseases. 2. Circulatory Disorders related to Skin Diseases. 3. Sexual Disorders related to Skin Diseases. 4. Anemia as related to Skin Diseases. 5. Malaria as related to Skin Diseases. 6. Syphilis as related to Skin Diseases.

*Lecture IV.*—1. Diet as related to Skin Diseases. 2. Hygiene as related to Skin Diseases. 3. General Therapeutics in Skin Diseases. 4. Frequent Importance of Skin Lesions, as indicating Systemic Diseases. 5. Importance of Correct Local Treatment.

Rarely has so much information of value been imparted in so small a book, and never before have the relations of skin disorders to other maladies been explained with such lucidity. It is a monograph that no practising physician can afford to do without.

**Ellis's Demonstrations of Anatomy.** Being a guide to the knowledge of the human body by dissection. Twelfth edition. Revised and edited by **Christopher Addison,** M.D., BS. (Lond.), F. R. C. S. Lecturer in Anatomy, Charing Cross Hospital, Medical School, Examiner in Anatomy, Royal College of Surgeons, England, etc. Illustrated by 306 engravings on wood, of which a large number are in colors. Octavo volume, 861 pages. New York: William Wood & Company. 1906. (Price, muslin binding, $3.50 net).

Without doubt the best way to obtain an accurate knowledge of human anatomy is through dissection of the various structures of the body. To do this work properly the anatomical student must have a good instructor and an excellent book as a guide. Ellis's demonstrations for many years have been considered among the best, having stood the test of experience. Addison has revised the present edition to meet the advances in the knowledge of anatomy and the changes in the methods of teaching. The material has been rearranged to follow the course taken by students; in appropriate places old matter has been eliminated, principally in those relating to the viscera; and sixty-two illustrations, twenty-seven of which are in colors, have been added. The marginal references are a great convenience to the student of anatomy, and the book is in every respect worthy of continued confidence and favor.

---

**A Treatise on the Diseases of Infancy and Childhood.** For Students and Physicians. By **Henry Koplik**, M.D., Pediatrist to Mt. Sinai Hospital, ex-President American Pediatric Society, etc., New York. New (2d) edition. Revised and enlarged. Octavo, 868 pages, 184 engravings and 33 plates. Lea Brothers & Co., Publishers, Philadelphia and New York, 1906. (Cloth, $5.00; Leather, $6.00 net).

Koplik is not a new name nor is he a new author to become acquainted with. His first edition was received with encomiums and his fame as an accomplished pediatrist is world-wide. His treatise takes rank with the best in any land or tongue and his ability as a teacher is acknowledged everywhere.

In this edition the advances of the past few years have been met, and the author's vast personal experiences have been incorporated, thus making the work a practical clinical treatise. It becomes, therefore, of extreme value to the general practitioner, while the specialist of necessity will appreciate and possess it. The section on infant feeding, in which every young mother is so deeply interested, is well worth the price of the book; it is scientifically sound and intensely practical. A strong feature of the treatise is the importance given to physiology and pathology of the newborn; the specific infectious diseases, too, have been elaborately dealt with; in short, the author has presented a detailed yet succinct reflection of the present status of pediatric knowledge, which is not surpassed by any other treatise on the subject. The illustrations are superb, many of them being original. The plate delineating Koplik's spots, a pathognomonic sign of measles, is deserving of special commendation. The volume is provided with an excellent index, which is an essential of every medical treatise.

---

**The International Medical Annual.** A yearbook of Treatment and Practitioner's Index. Twenty-fourth year. Substantially bound in cloth and fully illustrated by plates in color and black and white. New York: E. B. Treat and Company. 1906. Octavo, 700 pages. (Price, $3.00 net, post or express paid).

This book is always welcomed by those most familiar with its scope and purpose. It is a résumé of the medical literature of the past year, prepared by thirty-one department editors, and contains added articles by noted specialists. The staff of editors is made up of men with established reputations in their respective fields,—all specialists of the highest type.

This is the twenty-fourth annual issue of this work and to its credit, its general appearance, size of page, and form remain unchanged. This uniformity in its external fashion gives the volumes a fine showing in the library. Its dictionary make-up, supplemented by a full index, makes it a most complete and satisfactory year book, and with it the busy practitioner is enabled to keep himself in touch with the most recent advances in knowledge and practice respecting any subject in medicine or surgery.

The present number exceeds that of any of its predecessors, as might be expected, and it is not second to any work of its kind in the world.

---

**The Opthalmoscope and How to Use It.** By **James Thorington,** A.M., M.D., Professor of Diseases of the Eye in the Philadelphia Polyclinic; Ophthalmologist to the Elwyn, Vineland, and New Jersey State Training Schools for Feeble-minded Children, etc. 12 colored plates and ther illustrations. 8 vo. pp. 398. Philadelphia: P. Blakiston's Son & Company. 1906. (Cloth, $2.50 net).

Thorington has become well known to the medical profession as an author of scientific attainments, through his previous works, "Refraction and how to Refract," and "Retinoscopy." In the present work he discusses the use of the most important diagnostic instrument relating to the eye that the last half of the nineteenth century produced.

The author gives the most perfect directions for the employment of the ophthalmoscope in the most concise manner, so that even the neophyte cannot fail to comprehend the correct use of the instrument. Moreover, he tells what we should see in the eye, both in health and in disease. Not only this, but he tells how to refract, discusses optics, instructs in retinoscopy, and informs the student upon many cognate subjects.

Thorington, however, has limited his descriptions in the main to those diseases which interest in particular the general practitioner in his daily work. It is not intended to be an exhaustive treatise but, instead, a description of the ophthalmoscope and how to use it,—a purpose in which the author has succeeded in a most admirable manner. The drawings, half-tones, and colored plates give excellent illustration of the text and make it clearly as well as easily understood.

---

**A Textbook on the Practice of Gnyecology.** For Practitioners and Students. By **W. Easterly Ashton,** M.D., LL. D., Fellow of the American Gynecologic Society; Professor of Gynecology in the Medico-Chirurgical College of Philadelphia. Second Edition, revised. Octavo of 1079 pages, with 1046 original line drawings. Philadelphia and London: W. B. Saunders Company. 1906. (Cloth, $6.50 net; Half-Morocco, $7.50 net).

It is a rare occurrence for the second edition of a medical work to be required within six months from the appearance of the first, but this distinction belongs to Ashton's treatise. It is not only unique in this respect but further, because it describes in adequate detail what is to be done and tells precisely how to do it. Still further, the engravings are line drawings from living models, dissections on the cadaver, actual apparatus, and the operative technics of other authors,—ten-hundred and forty-six cuts in all.

The demand for this edition, coming so soon after the original issue of the book, has left very little to be done in the way of revision. Some errors have been corrected and a few of the illustrations have been altered,—otherwise the book remains the same. What we said, therefore, in commending the first edition remains good for this one—namely that it is a practical treatise by an accomplished gynecologist.

**The Physical Examination of Infants and Young Children.** By **Theron Wendell Kilmer,** M.D., Adjunct Attending Pediatrist to the Sydenham Hospital; Instructor in Pediatrics in the New York Polyclinic Medical School and Hospital; Attending Physician to the Summer Home of St. Giles, Garden City, New York. Illustrated with 59 Half-tone Engravings. 12mo., 86 Pages. F. A. Davis Company, Publishers, Philadelphia, Pa. (Price: $.75 net).

This author has done the medical profession much service in presenting a subject not heretofore dealt with in monographic form. Whatever has been written regarding it, is to be found in textbooks or general treatises on diseases of children, hence lacks the emphasis that attaches to the treatment of the topic in a separate book, as in the present instance. Kilmer displays an intimate knowledge of children and how to handle them; he knows how to interpret not only their symptoms but their mannerisms, and he has told the story most attractively as well as in thorough scientific fashion. The illustrations, practically all original, are superb and bring out the salient features of the text with insistence. It is, by and large, a book that will do a vast amount of good to all who treat diseases of children

---

**Saunders's Question Compends.** Essentials of Materia Medica, Therapeutics, and Prescription Writing. By **Henry Morris,** M.D., College of Physicians, Philadelphia. Seventh edition, thoroughly revised. By **W. A. Bastedo,** Ph.G., M.D., Instructor in Materia Medica and Pharmacology at the Columbia University (College of Physicians and Surgeons). New York. 12mo, 300 pages. Philadelphia and London: W. B. Saunders & Company, 1905. (Cloth, $1.00 net).

A good way to study the topics of which this excellent little volume treats is to use it as an index and pursue the investigation from that basis. The student always has needed reminders such as this and will continue to do so probably as long as time and memory run.

This edition is made to conform to the eighth revision of the pharmacopeia. Articles have been added on diphtheria antitoxin, thyroid extract, and arganotherapy, while a few obsolete drugs have been dropped. This makes the book as complete as such a compend can be, and it is well worthy of the continued favor of those for whom it was particularly designed.

---

**A Compend of Medical Chemistry, Inorganic and Organic,** including Urinary Analysis. By **Henry Leffman,** A.M., M.D., Professor of Chemistry in the Woman's Medical College. Fifth edition, revised. Philadelphia: P. Blakiston's Son & Co. 1905. (Price, $1.00).

This author is a teacher of experience and knows what students most need to meet the requirements of the classroom and laboratory. He has prepared a compend of medical chemistry that has merited a fifth edition, hence has won a place for itself among those for whom it is especially prepared. We recognise no good reason why books of this kind,—quiz compends,—should not be commended, when they are based on scientific facts. Teachers of acknowledged standing write them and students want them. They have their place and no one presumes to give them standing outside their legitimate sphere. This is a good book and deserves to be so rated.

---

**Transactions of the American Laryngological Association.** Twenty-seventh Annual meeting, held at Atlantic City, N. J., June 1, 2, and 3, 1905. Dr. James E. Newcomb, secretary. Published by the Association. New York: Rooney and Otten Printing Co., 1905.

This association, one of the older of the special societies, always presents an interesting volume of transactions. An exhaustive article in this book on deflections of the nasal septum is presented by Dr. Otto T. Freer, which is a critical review of the subject, and is profusely illustrated. The other papers are of fine quality and the discussions are highly instructive, A message of congratulation to the venerable Manuel Garcia and his reply afford interesting reading. This staunch association is in a thriving condition.

